# Improving Tip Aesthetics Using Mastoid Fascia Tissue Graft in Rhinoplasty: A Reliable Alternative to Soft Cartilage Grafts

**DOI:** 10.1093/asjof/ojae007.029

**Published:** 2024-04-12

**Authors:** Paige Goote, Bugra Tugertimur, Shaishav Datta, Steven Hanna, Matthew Morris, David Mattos, Richard Reish

**Affiliations:** Northwell, Great Neck, NY; Long Island Plastic Surgical Group, New York, NY; Manhattan, Eye, Ear and Throat Hospital, New York, NY; Private Practice, Toronto, ON, Canada; NYY Langone, New York, NY; Long Island Plastic Surgical Group, New York, NY; Long Island Plastic Surgical Group, New York, NY

## Abstract

**Goals/Purpose:**

Traditional rhinoplasty tip grafts often lead to visibility issues, prompting patients to seek revision surgery. The mastoid fascia tissue graft (MFTG) provides a natural-looking alternative with fewer complications. MFTG remains less visible through the skin, enhancing long-term aesthetic results. This eight-year study on 193 patients examines MFTG's effectiveness in nasal tip refinement, evaluating revision and infection rates.

**Methods/Technique:**

A retrospective analysis of MFTG use for nasal tip refinement during open rhinoplasty in the senior author's practice was conducted from July 2014 to June 2022. Inclusion criteria encompassed open rhinoplasty cases using mastoid tissue for tip refinement with at least 12 months of follow-up. Among 2003 cases, 193 met these criteria and were evaluated for subsequent revision and infection rates.

**Results/Complications:**

The average patient age was 34.2 years (175 females, 18 males). Primary rhinoplasties were done on 114 patients, with 79 receiving revision surgeries. Average follow-up was 14.8 months. 6 patients (3.1%) needed extended antibiotics. Overall, 6 (3.1%) patients required revision rhinoplasty, comprising of 2 (1.8%) primary and 4 (5.1%) secondary rhinoplasty patients. These revisions were unrelated to the use of MFTG for nasal tip refinement.

**Conclusion:**

MFTG use for nasal tip refinement is associated with low revision rates for both primary and secondary rhinoplasty compared to rates in current literature, without increase in complications or prolonged operative durations. MFTG for nasal tip refinement is a safe and convenient technique delivering excellent aesthetic outcomes with minimal morbidity.

